# Dynamic Staffing and Rescheduling in Software Project Management: A Hybrid Approach

**DOI:** 10.1371/journal.pone.0157104

**Published:** 2016-06-10

**Authors:** Yujia Ge, Bin Xu

**Affiliations:** 1 College of Computer and Information Engineering, Zhejiang Gongshang University, Hangzhou, Zhejiang, China; 2 College of Information Engineering, Zhejiang University of Technology, Hangzhou, Zhejiang, China; Southwest University, CHINA

## Abstract

Resource allocation could be influenced by various dynamic elements, such as the skills of engineers and the growth of skills, which requires managers to find an effective and efficient tool to support their staffing decision-making processes. Rescheduling happens commonly and frequently during the project execution. Control options have to be made when new resources are added or tasks are changed. In this paper we propose a software project staffing model considering dynamic elements of staff productivity with a Genetic Algorithm (GA) and Hill Climbing (HC) based optimizer. Since a newly generated reschedule dramatically different from the initial schedule could cause an obvious shifting cost increase, our rescheduling strategies consider both efficiency and stability. The results of real world case studies and extensive simulation experiments show that our proposed method is effective and could achieve comparable performance to other heuristic algorithms in most cases.

## Introduction

Software project process is not a rigorous engineering process because scheduling schemes can be influenced by various dynamic elements including the skills of engineers, the growth of those skills, and cooperation in teams etc. It is difficult for software project managers to meet budget and schedule constraints set by its stakeholders. To solve this problem, researchers have developed several approaches to efficiently assign employees to tasks [1][2][3][4][5]. However, most resource-constrained scheduling techniques focus on the availability of resources instead of the resource productivity [6]. Significant productivity differences do exist among software developers. Therefore, to make a more realistic and reasonable schedule, productivity factors, such as learning, schedule pressure, should also be considered in software project management. When factors change or status becomes bad to projects, project control actions are taken and schedule must be revised to follow the change. To prevent ineffectual project control which is also the main cause of over-budget and behind-schedule projects [7], an efficient rescheduling approach needs to be carefully designed to make the project back to the track. However, the rescheduling problem is not emphasized sufficiently in the literature of scheduling models. To the best of our knowledge, the previous researches fall short of adequately explaining human capabilities to conquer the complex and dynamic nature of software project management.

Therefore, the main goal of our current work is to do the scheduling and rescheduling model in which optimal control strategies could be computed with reasonable complexity. In this paper, the following work related to our model and approach is reported.

1. Proposing a software project scheduling/rescheduling framework which supports dynamic staffing and rescheduling;
2. Applying a hybrid approach based on a Genetic Algorithm (GA) and Hill Climbing (HC) considering both efficiency and stability;
3. Conducting case studies and empirical studies.

## Related Work in Software Project Scheduling and Rescheduling

Our work is to investigate assigning employees to tasks and minimize the total project cost by stochastic search methods in project scheduling and rescheduling.

### Software Project Scheduling

Several researchers compare the results from heuristic and metaheuristic techniques when solving resource-constrained scheduling problems [8]. Heuristic approaches are typically preferred for solving large-scale problems. One of these techniques is GA, introduced in the 1970s by John Holland [9]. Ever since, GAs have been used by many researchers to study scheduling problems and its variations [10]. In software project optimization fields, stochastic search methods have also been widely used. A general introduction and survey of recent achievements in Search Based Software Engineering can be found in the work by Harman *et al*. [11] including search-based software project scheduling in which GAs are considered popular methods. Since no single GA approach can consistently perform best in all problems, different GAs should be designed and tuned for software project scheduling problems. Our previous task-based model can be considered an early effort to apply GAs in the software project management environment [1], much as timeline-based model [3] does. Similar to our previous work, Alba and Chicano [4] also apply GAs for automated task assignments, showing that GAs are flexible and accurate for software project scheduling, and function as an important tool for automatic project management. In their work, an in-depth analysis was performed with an instance generator, where 48 different project scenarios were solved in software project management. Few human resource factors were considered in their model. To achieve better performance in a realistic setting, a more sophisticated model is required. There are some more recent research works on software project scheduling problems. Ferrucci *et al*. [12] proposed a multi-objective decision support approach to help software engineers balance project risks and duration against overtime. They had extensive experiments to show their effectiveness of their approach. Ren *et al*. [13] presented an approach based on Cooperative Co-evolution to optimize both developers’ team staffing and work package scheduling to achieve early overall completion time which has different objectives from our research. The above works are all dealing with scheduling problems under software project circumstances. However none of them consider rescheduling problems during schedule execution.

### Rescheduling Methods

Rescheduling techniques are also proposed in the areas, such as job shop problems [14][15][16]. Nevertheless there is still very limited support under software projects circumstances. Sukhodolsky uses discrete optimization techniques for finding optimal control actions the manager should take to meet project’s deadline [17]. However, the situations in real projects may not be as simple as it is stated in the paper.

### Software Project Effort Estimation

The task model of our approach is also related to the precision of software project effort estimation. Among these estimation models, COCOMO [18] and its improvement COCOMO II [19] are the most commonly used effort estimation models. Other estimation methods, e.g., analogy-based estimation [20][21] and Bayesian analysis [22], also exist. Individual project effort estimation method using genetic programming [23] to predict the software development effort shows the accuracy results when these projects have been developed in a disciplined manner within a development-controlled environment. Most of the existing software task effort estimation methods could be employed before initiating the scheduling and rescheduling approach in this paper.

### Software Development with System Dynamics

Human resource factors play an important role in software development. For example, pressure from tight schedules can cause an employee to speed up work. System Dynamics is a method to model a system by using continuous feedback loops. Since the first application in 1991 of system dynamics by Abdel-Hamid [24] on project management, there has been additional extension work within the realm of project management, such as the system dynamics extension modules [25][26]. Besides the continuous modeling approach, other researches focus on discrete-event approaches [27][28]. Hybrid software process simulation models combining discrete event and system dynamics approaches are also introduced to support software project estimation and project management [29][30][31]. Penta *et al*. [32] formalize communication overhead and use a search-based project staffing and scheduling approach on two large real world maintenance projects.

As described in this paper, our model incorporates system dynamics to illustrate team productivity and use stochastic search methods to solve the optimization problem in scheduling and rescheduling, and has the potential to become a more realistic model for project managers to adopt.

### Project Scheduling Tools

There are many commercial project management tools such as *Microsoft Project* and *Symantec* Corporation’s *Time Line*. None of these, however, provides automatic scheduling functionality. An early effort on software to help automatic scheduling for project management is *Opensched* [33]. It reads a file describing the project as input and produces textural descriptions of the generated project plan, Gantt charts and network diagrams. The input includes tasks which must be accomplished, resources (e.g., people, equipment, and facilities) which may work on tasks and work that has already been completed. However, the model supported in *Opensched* is very simple. A tool named *IntelliSPM* [34] is provided to support software project management by Computational Intelligence considering significant human factors. Although it is not always practical to use automated project scheduling in project management, research is still needed for improving the overall capabilities of current tools which is also what we are trying in this paper.

## Dynamic Staffing and Rescheduling Approach

**Fig 1:**
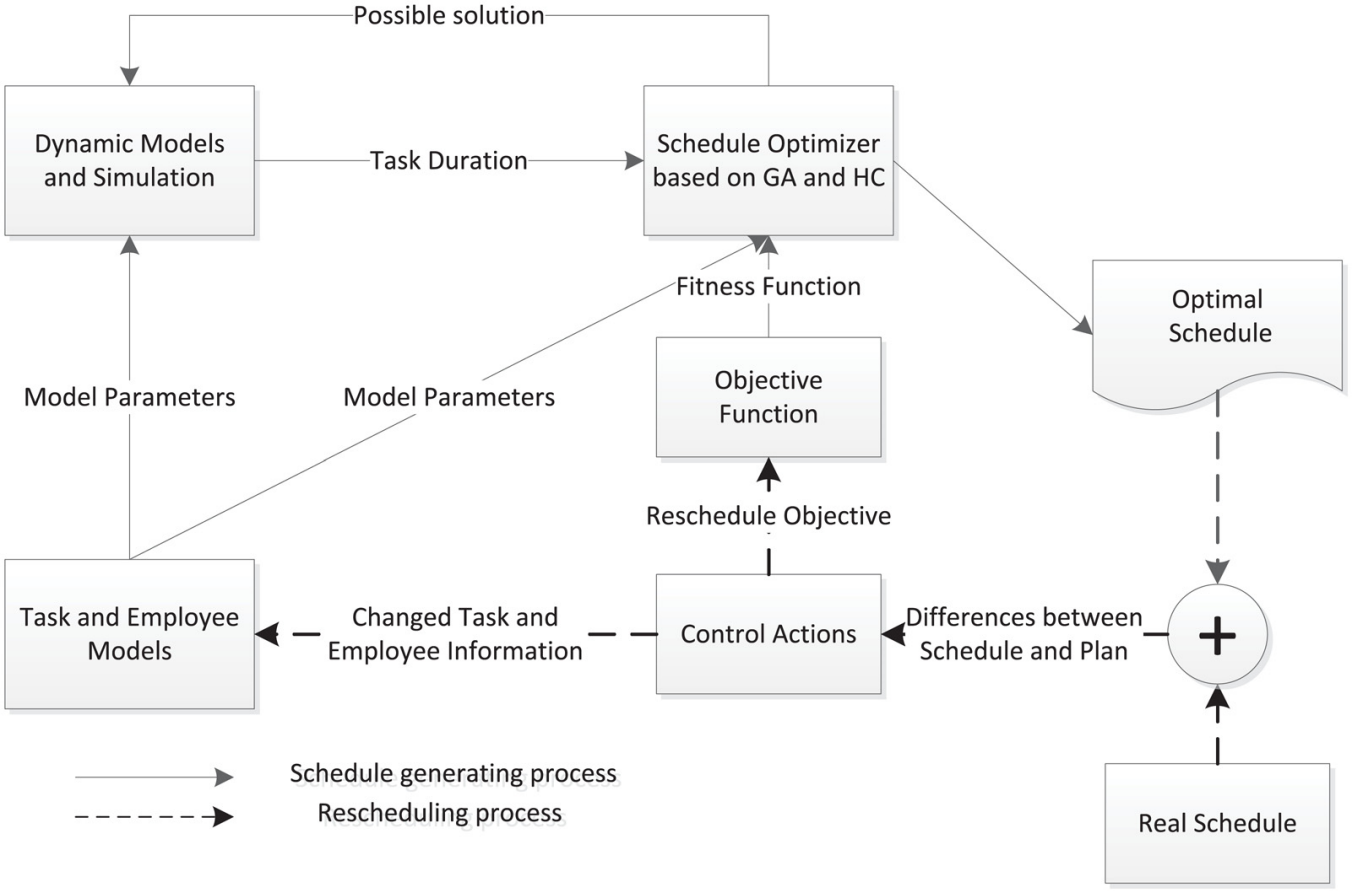
A Software Project Scheduling and Rescheduling Framework with Dynamic Factors.

Our proposed approach for team productivity modeling and schedule/reschedule optimization process is illustrated in Fig 1. In this framework, “Schedule Optimizer based on GA and HC” is the key component. It helps to generate the project schedule at the beginning of a project, or re-generate schedules when tasks/team members change or differences between execution of the project and the plan become big. The inputs of the optimizer include model parameters of detailed task and employee information, the duration of each task which is achieved by simulation, and the fitness function according to management objectives and control actions. “Task and Employee models” includes the static part of task models (i.e., task estimated effort, task penalty model and required task skill lists, etc.) and employee models (i.e., skill lists, payment model, etc.). Before a project execution, the “schedule optimizer” generates the initial optimal schedule according to the planned tasks and assigned employees. During the project execution, real project progress is compared to the initial plan. When control actions are taken by a manager, rescheduling happens and accordingly the model parameters are changed. Re-calculation will be done to generate a new schedule for the remaining project. The new generated schedule is evaluated by a “rescheduling” objective function considering stability and efficiency. The dashed lines illustrate the process involved in the project control activities.

### Task and Employee Models

Task and employee models include the information about the tasks of a project and employees assigned to this task. A project is represented as a Task Precedence Graph (TPG), an acyclic directed graph consisting of a set of tasks *V* = {*T*_1_, *T*_2_,…, *T*_*n*_} where *T*_*k*_ represents task *k* of the project, and precedence relations *P* = {(*P*_*ij*_); *i* < > *j*, 1 ≤ *i* ≤ *n*, 1 ≤ *j* ≤ *n*}, where *P*_*ij*_ = 1 if *T*_*i*_ must be completed before *T*_*j*_ starts, and *P*_*ij*_ = 0 if not. Associated with each task *T*_*k*_ is the estimated effort, required skills, and deadline. Project team members *E* = {*E*_1_, *E*_2_,…, *E*_*n*_} are the resources for the project where *E*_*k*_ represents employee *k*. Each *E*_*k*_ is associated with a list of skills he/she possesses with corresponding proficiency levels, salary rate, maximum workload (a limit to the amount of work load they can be assigned), and learning factor (a factor to reflect the improvement of their skill proficiency during working).

### Dynamic Models and Simulation

**Fig 2:**
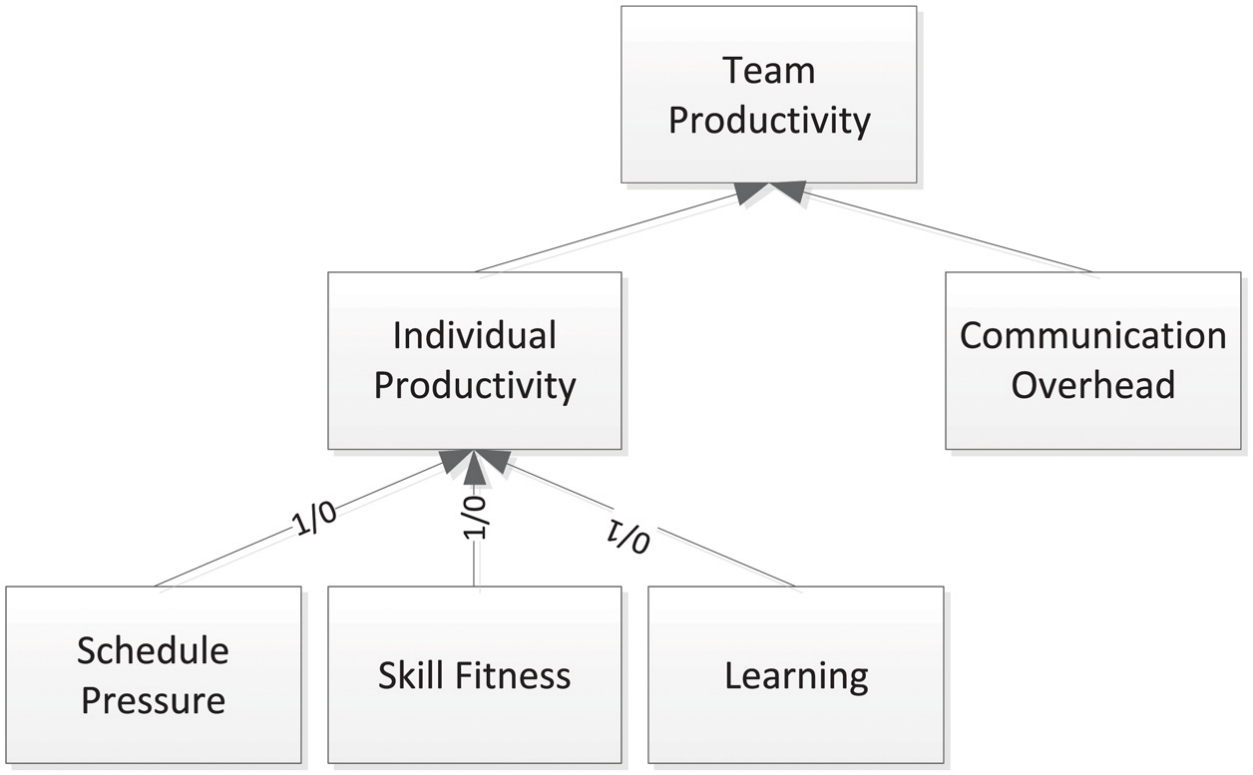
Team Productivity Model.

Team productivity determines the overall project performance in a software development process. Fig 2 models this key component and its related factors. “Individual productivity” and “communication overhead” are major factors contributing to the “team productivity”. “Individual productivity” is affected by “schedule pressure”, “skill fitness” and “learning” factors. Although other factors, such as employee motivation, are also critical to individual productivity, we will not completely include all of them in this paper for the purpose of demonstrating key concepts. These factors are also controllable by project managers through a control parameter with value 0 or 1 to be turned on or off.

In the psychological model of group productivity by Ivan Steiner [24], the productivity of the software development group is stated as: Actual Productivity = Potential Productivity—Losses Due to Faulty Process, where losses due to faulty process refers basically to communication and motivation losses. Similarly, team productivity (*P*_*team*_), i.e., the productivity of a team of people working on a given task in our work, is defined as the summation of individual productivity (*InP*_*i*_) affected by communication overhead factor (*ComOverhead*(*n*)) in Eq (1).

$$\begin{array}{c} P_{team}=\left(1-ComOverhead\left(n\right)/100\right)*\sum_{i=1}^{n}InP_{i} \end{array}$$ (1)

Abdel-Hamid and Madnick [24] demonstrated that communication overhead increases in proportion to *n*^2^, where *n* is the size of the team, which can be expressed as Eq (2). When there is only one member in a team, it is obviously no need on team communication. As the size of the team increases, so does communication overhead. When the team size exceeds *MaxSize*, it is assumed to have 100% communication overhead in our work.

$$\begin{array}{c} ComOverhead\left(n\right)=\left\{\begin{array}{cc} 100 & n>MaxSize\\ 100*{\left(n-1\right)}^{2}*/{\left(MaxSize-1\right)}^{2} & 2\leq n\leq MaxSize\\ 0 & n=1 \end{array}\right. \end{array}$$ (2)

Individual productivity is represented using Eq (3) which is affected by skill, learning, and schedule factor.

$$\begin{array}{c} InP=nomP*f_{skill}*f_{learning}*f_{schedule} \end{array}$$ (3)

where *InP* is the individual productivity; *nomP* corresponds to the nominal productivity which is generally the ideal individual productivity without considering factors such as schedule pressure and learning which is 1 by default; *f*_*skill*_, *f*_*learning*_, and *f*_*schedule*_ correspond to the skill fitness factor, learning factor, and schedule pressure factor and defined in Eqs (4)–(6) respectively.

$$\begin{array}{c} f_{skill}=\sum_{i=1}^{s}S_{i}/s \end{array}$$ (4)

Eq (4) is to evaluate an employee’s skill fitness to a task where *S*_*i*_ is the skill fitness level of the employee required for a given task and *s* is the number of skills required for a given task.

The learning curve has been studied for many years. Only a few papers, however, mention learning curve in Software Engineering, such as [24] where Eq (5) is adapted from. *f*_*learning*_ represents the improvement of understanding the task along with the progress of the task itself and is a S-curve equation

$$\begin{array}{c} f_{learning}=\left\{\begin{array}{cc} 1.6*\left(l_{i}-1\right)*X^{2}+1 & 0\%<=X<=50\%\\ 1.6*\left(l_{i}-1\right)*X+1.4-0.4*l_{i} & 50\%<=X<=75\%\\ l_{i}-3.2*\left(l_{i}-1\right)*{\left(1-X\right)}^{2} & 70\%<=X<=100\% \end{array}\right. \end{array}$$ (5)

where *X* is the percentage that a task has been completed and *l*_*i*_ is the learning property of an employee. To get *f*_*schedule*_, *schedule pressure* is defined as a function of the current time (*T*_*c*_) and the planned time (*T*_*p*_) in Eq (6) derived from the research by Abdel-Hamid and Madnick [24].

$$\begin{array}{c} \text{schedule}\;\text{pressure}=\left\{\begin{array}{cc} 5 & T_{c}>T_{p}\\ \min\{Workflow_{required}/Workflow_{normal},5\} & T_{c}\leq T_{p} \end{array}\right. \end{array}$$ (6)

where

$$\begin{array}{c} Workflow_{required}=effort_{remaining}/\left(T_{p}-T_{c}\right) \end{array}$$ (7)

$$\begin{array}{c} Workflow_{normal}=P_{team} \end{array}$$ (8)

*f*_*schedule*_ can then be obtained from *schedule pressure* using a lookup table from the Vensim documents [35] shown in Table 1. For example, if an employee’s individual productivity is 100 LOC/day, then his productivity is 100 * *f*_*schedule*_ which will range from 100-150 LOC/day, 100 being without schedule pressure.

**Table 1:** Lookup Table for *f*_*schedule*_.

*schedule pressure (x)*	*f*_*schedule*_
0 ≤ *x* < 1.1	1
1.1 ≤ *x* < 1.35	1.2
1.35 ≤ *x* < 1.75	1.4
1.75 ≤ *x* < 3.5	1.45
3.5 ≤ *x* ≤ 5	1.5

### Objective Function for Scheduling/Rescheduling

Our objective function is set to be the minimum total cost for performing the whole project. Several assumptions have been made for our scheduling problem: (1) Tasks cannot be interrupted and resumed; (2) Different people can work on different tasks at the same time, but cannot do work over their maximum overwork level; (3) Every employee assigned to a task needs to do the work for the whole duration for each task. When rescheduling, other than the efficiency factor (i.e., the total cost of project execution), the stability factor also need to be considered since the cost of changing staffing profile could be high and managers are in favor of rescheduling strategies addressing continuity in practice.

$$\begin{array}{c} \text{The}\;\text{objective}\;\text{function}=Efficiency*W_{e}+Stability*W_{s} \end{array}$$ (9)

Our scheduling/rescheduling objective function considers both efficiency and stability is shown in Eq (9). *W*_*e*_ and *W*_*s*_ are the weights for the efficiency and stability factors controlled by project managers which are determined by their needs and their experiences. *Efficiency* and *Stability* are calculated by Eqs (10) and (11) respectively. In generating an initial project plan, the stability factor is set to 0. During the rescheduling process, if a newly generated schedule radically different from the initial one can produce a great cost in changing staffing profile, the stability factor could weight more.

$$\begin{array}{c} Efficiency=1/Cost_{norm} \end{array}$$ (10)

where *Cost* is computed by the labor rates of each resource and the hours applied to the tasks of a schedule. *Cost*_*norm*_ is achieved by dividing *cost* by the maximum cost in the population of a GA.

Stability in Eq (11) is applied to minimize the impact of disruptions induced by the new schedule or introduced by new team members.

$$\begin{array}{c} Stability=1/StabilityPenalty_{norm} \end{array}$$ (11)

where *StabilityPenalty*_*norm*_ is achieved by dividing *penalty for stability* by the *maximum penalty for stability* in the population of a GA. Two kinds of stability are recognized, i.e. ex post stability, ex ante stability [36]. Ex post stability is considered and Eq (12) [14] is adapted in our model. The value is achieved by adding starting time deviation and actuality penalty.

$$\begin{array}{c} StabilityPenalty_{norm}=\sum_{j\in B}\left(\right|t_{j}^{{}^{\prime }}-t_{j}\left|+k/\sqrt{t_{j}-T}\right)/n \end{array}$$ (12)

where *B* is the set of tasks that need to be rescheduled. They are the tasks that remained unprocessed in the initial schedule and still need to be processed under new circumstances. *n* is the number of tasks in *B*. *t*_*j*_ is the predicted start time of task *j* in the new schedule. $t_{j}^{{}^{\prime }}$ is the predicted start of job *j* in the initial schedule. *T* is the current time.

Project managers could control the rescheduling plan by changing the stability and efficiency weights. The results of the experiment given for different number of tasks to be rescheduled are shown in our previous work [37] in which we normalized the durations by dividing it by the maximum duration in each column. The outcome shows that the stability factor has more effect on the project when small number of tasks is to be rescheduled. It would encourage managers to adjust the stability factor when the rescheduled point is at the tail of the whole plan of the project.

### Scheduler Optimizer based on Genetic Algorithm and Hill Climbing

**Fig 3:**
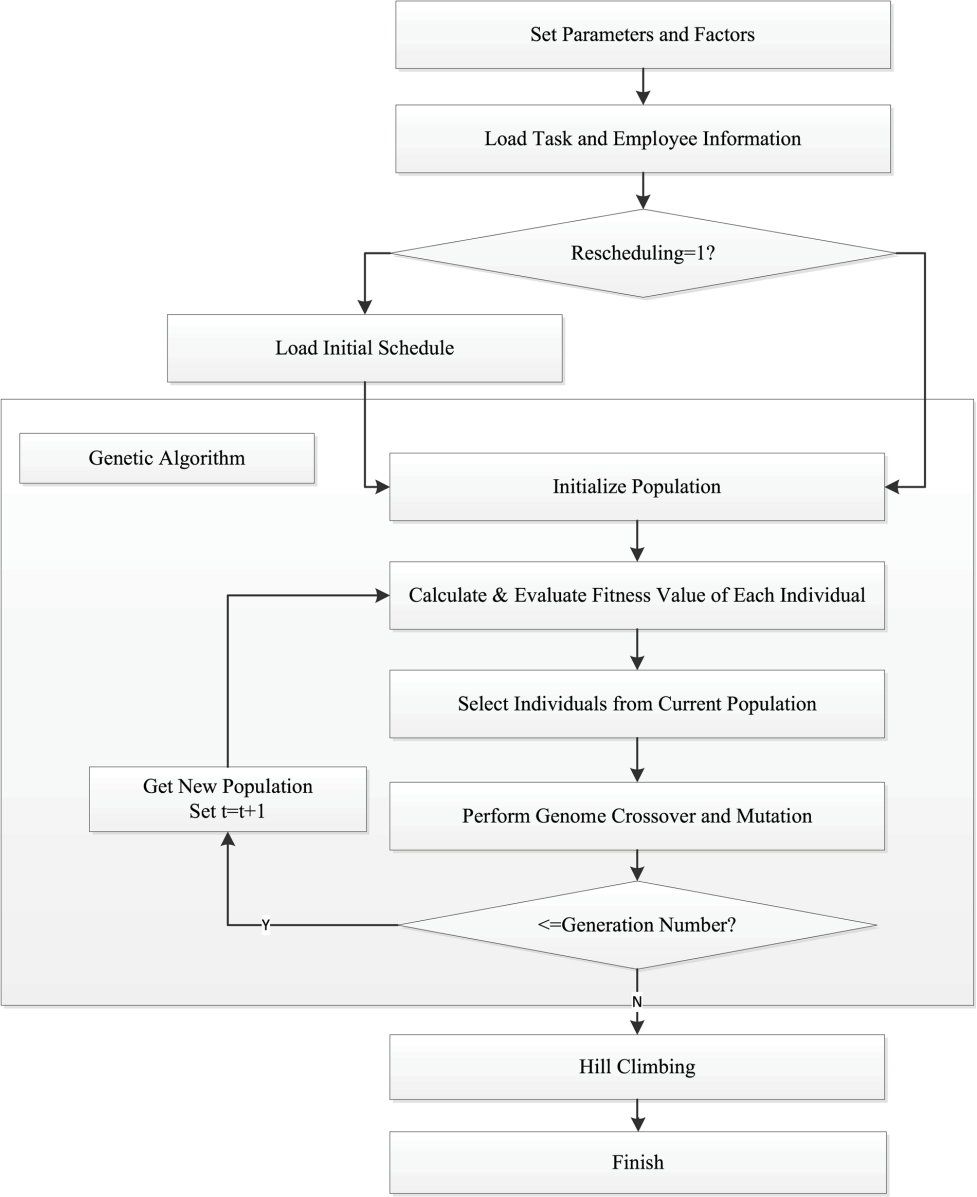
Process of Scheduler Optimizer.

With the GA’s ability on global optimization and the HC’s ability on local optimization, a hybrid algorithm combining GA with HC might be an ideal choice [3]. Based on the block theory, in the early stage of the GA computation, because there exist the many efficient, small blocks, under crossover operators, the probability that the small blocks can be united as big blocks are high. Therefore, the quality of the population is improved quickly. But in the later stage of GAs, when big blocks are becoming more and more similar, the efficiency of crossover operators is becoming much lower. At that time the quality of many individuals cannot be improved a lot which leads to low efficiency in the later stage of GAs. Therefore, we choose to use a GA at early stage followed by HC. The process of our optimizer is described in Fig 3. The first step is to set the parameters of the models, such as mutation probability, crossover probability, generation number, and population size of the GA. Then task and employee information is loaded into the GA. If it is a rescheduling process, the initial schedule plan also needs to be loaded. After these initialization steps, the GA runs until the generation number reaches the previously set one. Starting from the best individual generated from the last generation of the GA, HC runs to get an optimal schedule.

#### Genome representation

**Fig 4:**
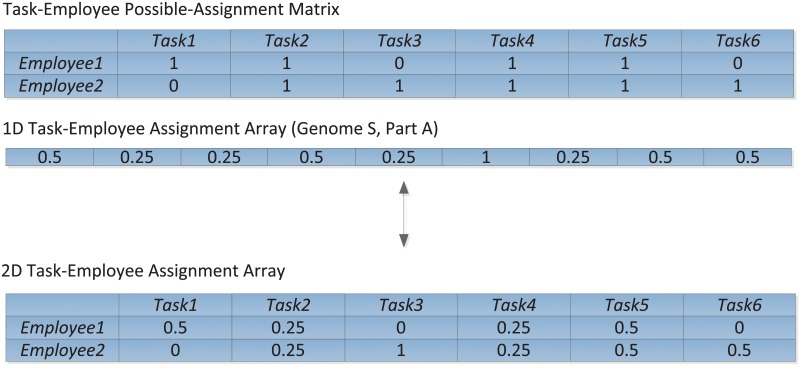
Part A of Genome S Generated from Possible-Assignment Matrix and 2D Task-Employee Assignment Array.

During the optimization, a candidate solution representation S is represented as {A, L}. Part A is a 1D task-employee assignment array that stores the information of task-employee assignments derived from 2D task-employee assignment array. For assigned tasks, an employee can work with a load of 0%, 25%, 50%, 75% or 100%. For example, 50% commitment means employee 1 can do 20 hours every week if he normally works 40 hours per week. A 2D task-employee assignment array can be squeezed into a 1D array according to the “possible” assignment matrix which is generated by the task-employee skill match. For example, in Fig 4, two employees are assigned to 6 tasks. Its task-employee possible-assignment matrix is derived according to their skill match, where 1 stands for possible assignment and 0 stands for no possible assignment. Since this possible assignment matrix is always stored in the model after the initial task-employee skill match calculation, the task-employee assignment 2D array could be squeezed into 1D array by taking out no-possible assignment elements (i.e., the “0” element). In this instance, there are 9 elements in task-employee assignment 1D array and the element order is the same as the order in task-employee possible assignment matrix in the row order.

Second part L is the priority list by which a certain topological-sort vector representing the execution order of the tasks in the schedule can be derived. Priority-based encoding, proposed by Gen and Cheng [38], can decide a certain order of tasks with information of tasks precedence information. Given a directed acyclic graph (DAG) *G* = (*V*, *E*), a topological sort is an order of all the vertex and for each (*u*, *v*)∈*E*, *u* appears before *v* on the list. Each DAG may have more than one topological sort. When there are two tasks competing for one position, the task with the higher priority wins. For example, for a project including 3 tasks, namely *T*_1_ to *T*_3_ where *T*_1_ is the starting task and it is the direct precedence of *T*_2_ and *T*_3_, the task priority list [2 1 3] means *T*_3_ whose priority value is 3 has higher priority than *T*_2_ with value 1. A topological sort {*T*_1_, *T*_3_, *T*_2_} can be generated which satisfies task precedence relationship. Using task order as genome directly in our scheduling problem could generate invalid individuals which dissatisfies the DAG. But priority-based sort only manipulates the priority information which must combine with project DAG information to be interpreted as a project execution. Therefore, no invalid individual will be generated.

#### Genetic operators

**Fig 5:**
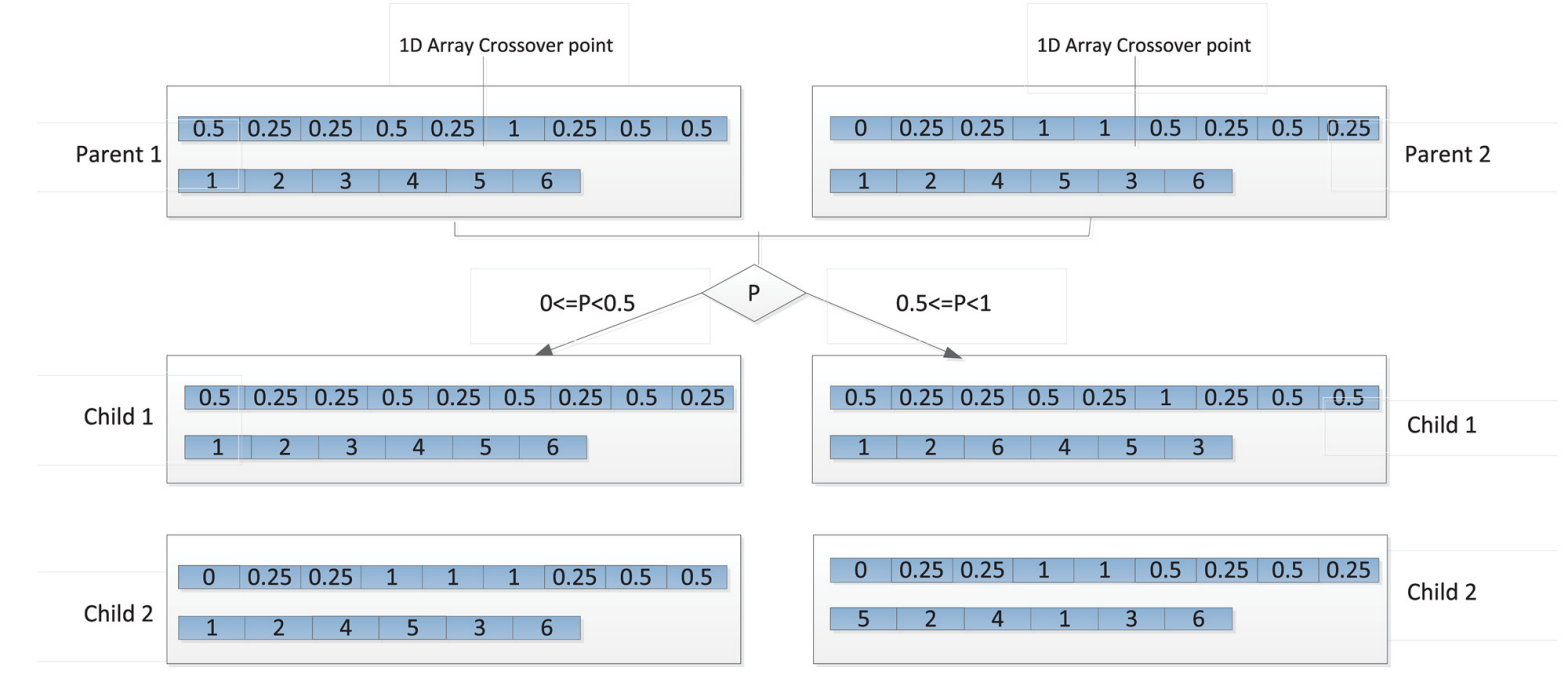
Crossover Operation of Genome S.

To manipulate the two structures of candidate solution S, the following operators are chosen. The initialization operator randomly chooses any value from possible values (0, 0.25, 0.50, 0.75, 1) to be the allele (i.e., possible settings for an attribute of an individual) of the initial population of 1D task-employee array (A). The priority list (L) is initialized as the random order from 1 to the total number of tasks. When rescheduling is necessary, tasks and employees with changed profiles are updated first and then the previous schedule is set as the initial population of a new GA calculation. The crossover operator of S invokes the crossover operator for each of the genomes in the composite genome according to a random number *P* as illustrated in Fig 5. The standard one-point crossover function is applied in part A of the genome and Order Crossover is used in part L. Because the two structures of the genome representation are independent and the 1D array genome is derived from the possible-assignment matrix, the offsprings of the crossover operator are always valid.

**Fig 6:**
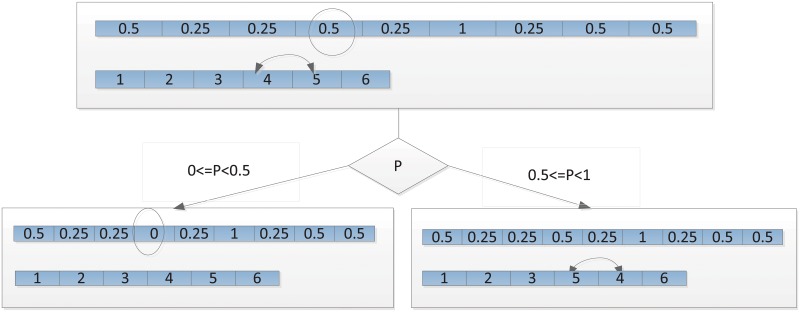
Mutation Operation of Genome S.

According to the mutation probability, we randomly select one of the two genomes to do the mutation shown in Fig 6. In the 1D array structure, we only change the certain elements while the certain elements are swapped in the task priority vector.

As stated in the subsection on genome representation, no invalid individual will be generated since operators only deal with possible task-employee assignment and priority task information.

#### Fitness function calculation

**Fig 7:**
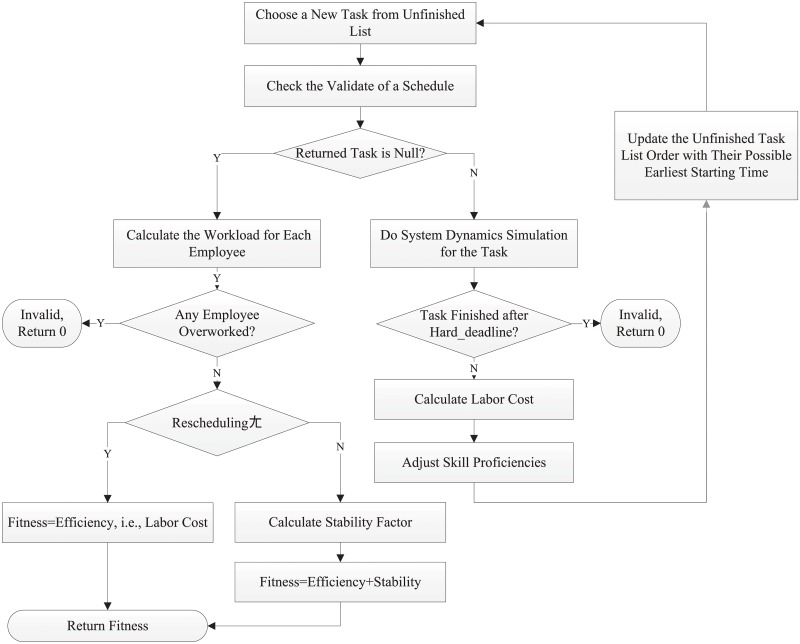
Fitness Function Calculation.

In our GA approach for software project scheduling, the fitness function calculation is the most complicated part. The detailed steps are illustrated in Fig 7: 1) Initialize the system by loading a task-employee assignment; 2) Set the number of the time unit; 3) Get the next task from a topological sorted list; 4) Check whether the task can start in this time unit or not by validating that all the; precedence tasks have been finished, all the employees are available, and all the employees do; not work over limit, if not, go to 2); 5) Do system dynamics simulation for each task; 6) At the end of execution of every task, calculate the cost, penalty and update the employee’s; overall experience and certain skill proficiency; 7) If all the tasks are finished, return the fitness score; 8) Start another loop from 2).

#### Hill climbing

Usually HC is much faster than GAs. However, the landscape in our problem has many local optima which makes HC difficult to achieve the global optimum. Therefore, in our algorithm, HC starts right after the end of our GA calculation. The HC algorithm that we use is that the best one is chosen as the start point. By using that best one, it is mutated at a randomly chosen single locus and the fitness is evaluated. If the mutation leads to a higher fitness, the new one replaces the old one. The procedure continues until the optimum is found.

## Experiments

**Fig 8:**
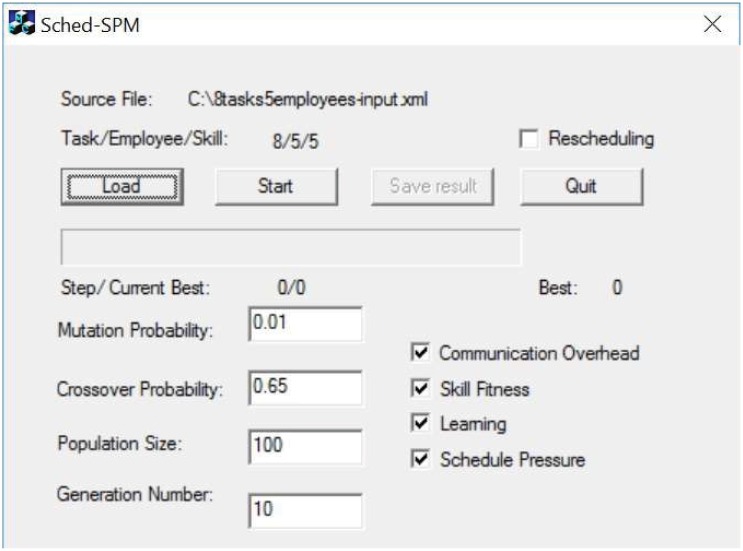
User Interface of *Sched-SPM*.

A preliminary tool for our hybrid staffing and rescheduling model was implemented in C++ with GALib [39], an open-source toolkit of Genetic Algorithms in various platforms including Unix and Windows. The software *Sched-SPM* is available at http://sched-spm.sourceforge.net. The graphical user interface of *Sched-SPM* is shown in Fig 8. The input and output files of the software are required as XML format. The input file includes tasks’ and employees’ information according to predefined file format. The output XML file of the generated schedule is accordance with Microsoft Project 2010 and could be open and edited within it. The software runs on a Windows environment with 2.9GHz processor, 8G RAM. Several experiments were conducted under this setting to evaluate the performance of the model.

### Parameter Settings

Genetic Algorithms are non-deterministic and factors such as the population size, generation number, mutation probability and crossover probability not only influence the time required to perform the GA algorithm, but also affect the quality of the result [3]. Several project simulation based tests were conducted to tune these parameters. Population size and generation number are set to 1000 and get the best results. It is reasonable to expect these two parameters set to 1000 to get good results for later larger and extensive experiments, although larger population size and generation number could result in better performance overall. The preliminary experiment results also show that the crossover probability set to 0.01-0.8 does not have a great impact on the performance of GAs. Hence, the crossover probability is set to 0.65 as our previous work [1][3]. The comparison of results by different mutation probabilities suggests that small mutation probabilities produce better results than the larger ones under some scenarios. Such a phenomenon is not unique [40]. This often occurs because higher mutation probabilities produce a greater percentage of not-so-good offspring. As the scheduling problem has many restrictions, it is easy to produce such kind of not-so-good offsprings by random mutation. In our experiments, mutation probabilities between 0.001 and 0.05 produced the best results. Accordingly, the default mutation probability is set to be 0.01 for the remainder of our work. To tune the value of *k* in Eq (12), experiments have been done in [37] where *k* is set to be 10, 100, 1000 and 10000 respectively. Stability Factor arrives from 0, 0.25, 0.5, 0.75 and 1. When stability factor weight more, the reschedule will favor on the schedule which is more similar to the initial plan and it will cause generated schedule with longer duration. We also expect that stability factor should increase steadily as the stability factor weights more. By this objective, *k* with 1000 seems more reasonable as the project includes about 12 tasks. When *k* is equal to 10 and 100, the line does not visibly going up which means *k* is too small to show the affect. As a result, we can consider *k* = 1000 outperform others.

### Case Studies

One experiment is a small project from Boyuan Software Company (www.139erp.com) who commits to the development of mobile phone sales management software and provides corresponding services. This project is to develop a mobile phone sales management on JAVA. It is extracted from a real project and some parameters such as learning, max workload, are redefined for incorporating our case study. The project consisted of 19 tasks and 9 employees with the properties listed in Tables 2 and 3 respectively. The employees who are all available from Jan 1, 2013 to July 1, 2013, in turn, each possessed 6 skills (JAVA programming language, testing, analysis/requirements, design, SQL server, domain knowledge) to a greater or lesser extent. It is not immediately obvious, even to the most experienced software manager, what the optimal assignments would be in this case.

**Table 2:** Task Properties of a Project with 19 Tasks and 9 Employees.

Task No.	Estimated Effort	Deadline	Precedence Tasks	Required Skills
1	0.3	Feb-01		3 6
2	0.6	Feb-01	1	3 5 6
3	0.8	March-01	1	3 5
4	0.3	March-01	1	3 6
5	0.5	March-01	3,4	3 4
6	0.4	March-0	1 2,5	3 4
7	0.4	Apr-01	2,3,5	6 4 5
8	0.4	Apr-01	6	3 4 5
9	0.7	Apr-01	4	6 4
10	0.5	Apr-01	7	4 5
11	0.5	Apr-01	8	1 3 5
12	0.4	Apr-01	9,10	1 6 4
13	0.5	May-01	7, 8, 9	1 2 6
14	0.8	May-01	11	1 4 6
15	0.5	May-01	13	1 4 5
16	0.5	June-01	12	1 2 5
17	0.2	June-01	14	1 2 6 4
18	0.2	July-01	14, 15	4
19	0.5	July-01	16,17,18	2 5 6

**Table 3:** Employee Properties of a Project with 19 Tasks and 9 Employees.

EmpID	Hourly Salary	Max Workload	Learning	skill1	skill2	skill3	skill4	skill5	skill6
1	38	110%	1.5	3	4	0	5	5	4
2	33	120%	1.3	4	0	2	0	5	0
3	28	120%	1.3	0	4	2	0	3	4
4	35	150%	1.1	4	0	3	3	0	5
5	40	120%	1.1	5	5	0	0	5	4
6	30	110%	1.3	0	0	4	4	0	3
7	30	110%	1.1	0	0	3	4	5	0
8	35	110%	1.2	5	0	4	5	0	0
9	40	100%	1.5	0	3	5	0	0	5

**Scheduling** The best result generated from the search algorithm is shown in Table 4. The cost of the schedule is 119358 RMB which involving all the dynamic factors. Table 5 shows the calculated costs with certain factors excluded. Without the learning factors or communication overhead, the overall cost increases as expected. Without schedule pressure, no solution is found in this highly constrained case. Although these results are intuitive in this simple scenario, in a more complicated situation data can help to analyze the influence of certain factors and to assist managers to make more sensible decisions.

**Table 4:** Generated Best Schedule from Our Algorithm.

Task ID	Days	Start Date	End Date	*Resource*
1	13	Jan 1	Jan 13	Employee9
2	21	Jan 14	Feb 3	Employee3[50%]
3	24	Feb 4	Feb 27	Employee7, Employee3
4	19	Jan 14	Feb 7	Employee9[50%]
5	18	Feb 28	Mar 17	Employee3[25%], Employee7[75%]
6	6	Mar 18	Mar 25	Employee6
7	24	Mar 18	Apr 10	Employee1[50%]
8	11	Mar 26	Apr 5	Employee7[75%]
9	15	Mar 18	Apr 1	Employee4
10	12	Apr 11	Apr 22	Employee1
11	18	Apr 6	Apr 23	Employee2
12	18	Apr 23	May 10	Employee1
13	29	May 3	May 31	Employee5, Employee1[25%]
14	24	Apr 24	May 17	Employee4
15	12	Jun 1	Jun 12	Employee1
16	16	May 16	May 31	Employee5
17	9	May 18	May 26	Employee1[75%]
18	5	Jun 13	Jun 19	Employee7
19	12	Jun 20	Jul 1	Employee1

**Table 5:** Results without Considering Some Factors.

Cost(RMB)	Best	Worst	Mean
With all the factors	119358	125759	121104
Without learning factor	131432	142992	141074
Without communication overhead factor	120202	128466	125936

**Fig 9:**
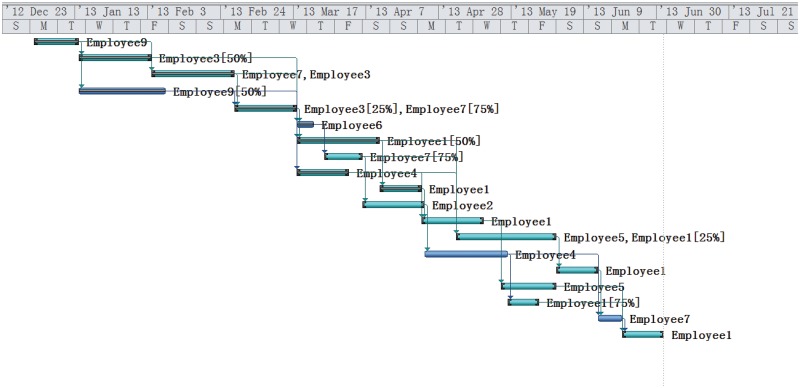
Initial Schedule and Project Execution.

**Rescheduling** On Apr 3, T1, T2, T3, T4, T5, T6, T7, T10 have been completed and other tasks have not yet been initiated. The corresponding Gantt graph of the initial schedule and project execution is presented in Fig 9. The project is greatly behind the plan notified by real execution data.

To bring any remaining project tasks into alignment with the planned schedule, managers have various project control actions to take, such as, adding people to the project, extending the time to completion, cutting out non-essential or less essential requirements. If option 1 is taken, genetic algorithm can easily generate a new schedule with the updated employees’ information and the updated tasks’ information if any new estimation has been made. If option 2 is used, it is just right-shift rescheduling and no genetic algorithm calculation needs to be done. If option 3 is taken, our model still easily fits by updating original task information tables. The result going after option 1 is shown as follows. Suppose manager would like to add another engineer (as shown in Table 6) into this team to catch up the schedule.

**Table 6:** Newly Added Employee’s Information.

EmpID	Hourly Salary	Max Workload	Learning	skill1	skill2	skill3	skill4	skill5	skill6
10	32	150%	1.5	3	4	0	0	5	5

Our rescheduling approach is applied for the remaining tasks that have not been started and Table 7 shows the comparison from the best newly generated schedule versus the initial schedule. *t*_*start*_ lists the start time of a specific task and d means the duration of this task. Rescheduling approach produced acceptable results both with stability (*W*_*s*_ = 0, *W*_*e*_ = 1) and without stability (*W*_*s*_ = 1, *W*_*e*_ = 1).

**Table 7:** Generated Schedule versus Initial Schedule.

Task ID	*t*_*start*_ in initial schedule	d	*t*_*start*_ in reschedule under Ws = 0, We = 1	d	*t*_*start*_ in reschedule under Ws = 1, We = 1	d
T8	Mar 26	11	Apr 3	10	Apr 3	12
T11	Apr 6	18	Apr 3	16	Apr 3	18
T12	Apr 23	18	Apr 3	10	Apr 3	11
T13	May 3	29	Apr 4	25	Apr 5	30
T14	Apr 28	24	Apr 29	18	May 5	19
T15	May 22	12	Apr 29	31	May 5	31
T16	May 16	16	May 19	11	May 24	12
T17	Jun 7	9	May 30	24	Jun 6	24
T18	Jun 13	5	May 30	9	Jun 6	10
T19	Jun 20	12	May 30	29	Jun 18	14
Duration (days)		130		121		136
Cost (RMB)		119358		128352		129327

The efficiency performance is affected by the stability measure. We can also see from the table that the schedule (*W*_*s*_ = 0, *W*_*e*_ = 1) has better efficiency performance over the schedule (*W*_*s*_ = 1, *W*_*e*_ = 1).

### Discussion via Management Experts’ Opinion

**Fig 10:**
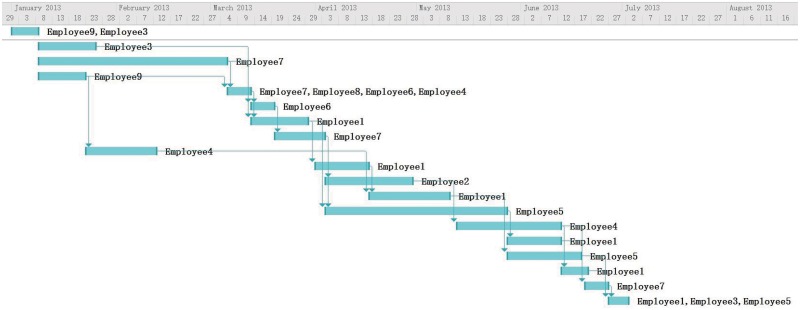
Scheduling Result from Expert 1.

**Fig 11:**
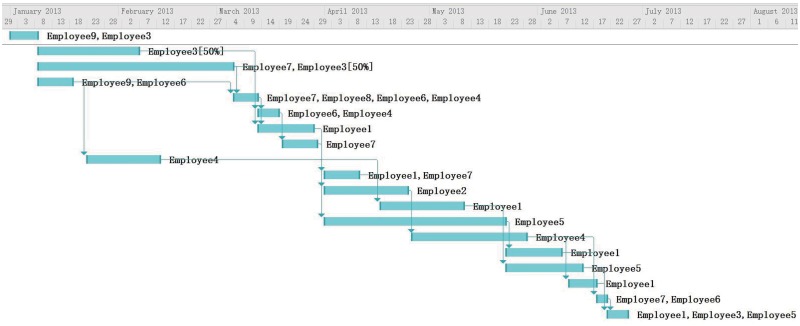
Scheduling Result from Expert 2.

**Fig 12:**
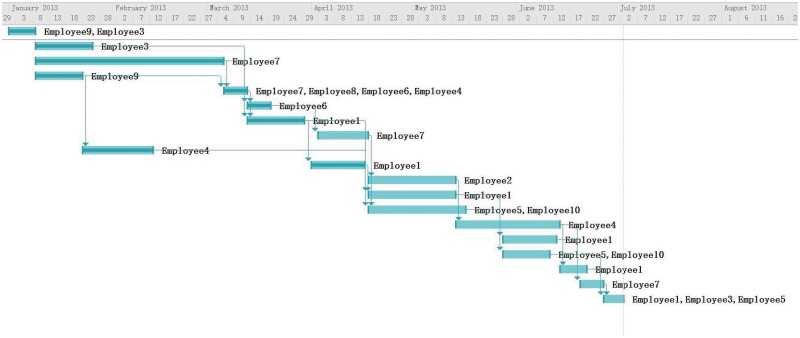
Rescheduling Result from Expert 1.

**Fig 13:**
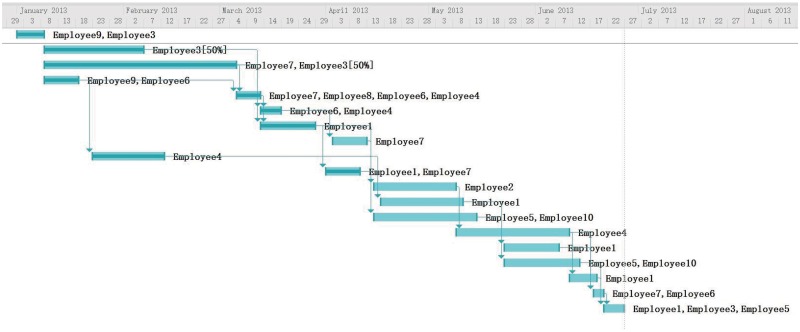
Rescheduling Result from Expert 2.

We decided to compare our model with experts’ opinion to evaluate the performance. We invited two senior software project managers to assign the employees to tasks. Both of them are experienced software project manager. One has 20 years in managing software projects in IT department of an international bank. The other worked on software development for almost 20 years and over 15 years on planning and managing projects in a country-wide e-commerce company. The experts shared the same assumptions as our model does and the overall objective is to have lest cost. But since humans could not calculate the schedule under all the assumptions set in the problem, they have their own inclination when assigning employees to tasks. For example, one manager has more concerns on skill matching of the employees and communication overhead. One pays more attentions on the overall duration of the project. Based on their work shown in Figs 10 and 11, we obtained cost of 124580 and 128030 respectively. The average time the experts spent developing the assignments was 3 hours. The average run time for our program is 30 minutes. The average cost of the experiments was slightly below the experts’ results. This is because the complexity of the problem makes humans difficult in achieving a schedule with minimal cost. When doing the rescheduling, experts change the schedules based on their previous plans. By adding Employee 10 to Task 13 and Task 16, the task duration is shorten to catch up the overall project duration. The results in Figs 12 and 13 are similar to our program’s result but the overall costs are higher. The experts are reluctant to change other tasks other than the tasks affected by the newly added employees. During real project execution, experts may consider more factors such as the personality and experience factors which are currently difficult to model.

**Fig 14:**
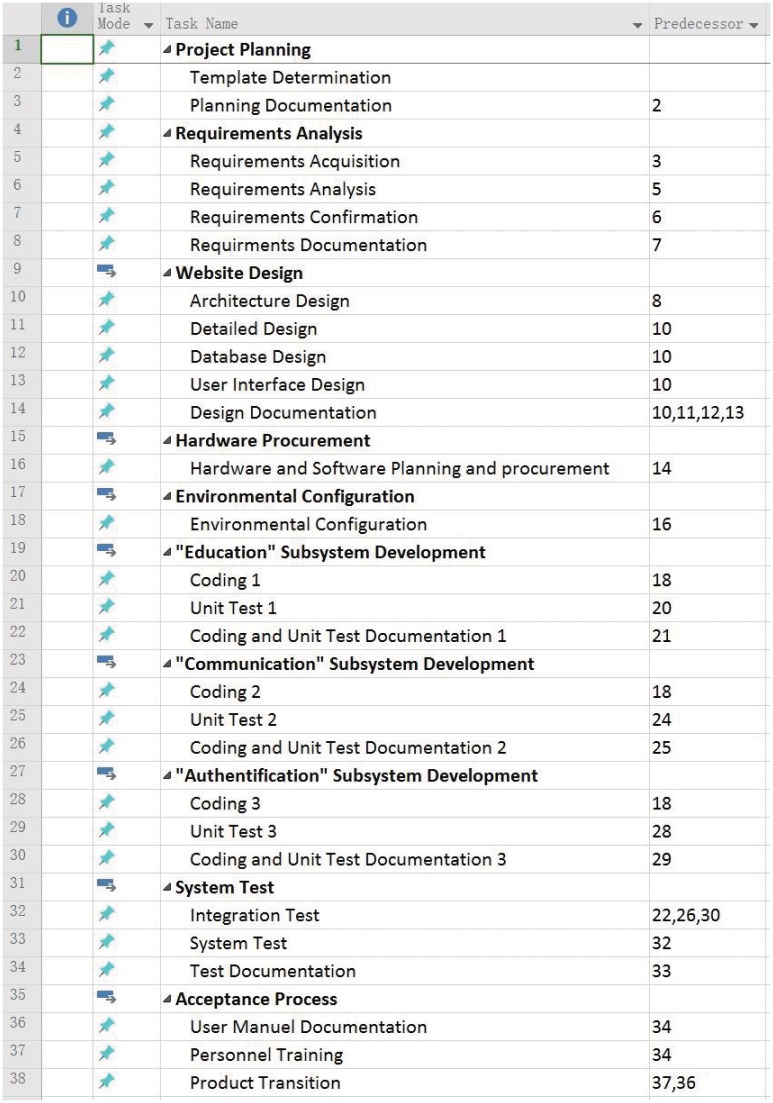
WBS of a Web Site Development Project.

The experts also work on planning for a college web site project which represents a common category of software projects. The project is to develop a system for students and teachers to communicate in JSP technology. There are 28 tasks, 8 kinds of skills, and 10 employees available for this project. Work Breakdown Structure (WBS) of this project is illustrated in Fig 14. Skill lists include Planning, Personnel Training, Documentation, System Design, Requirements Analysis, Maintenance and Testing. Experts took about 3.5 and 3 hours to get the results. Our program run 25 minutes to get a scheduling result. Similar to previous cases, the average cost of our approach is slightly less than experts’, i.e., 79000 and 77500 by experts respectively and 75800 by our program.

In conclusion, the schedules created by the experts were already acceptable but the results from the GA-HC program outperformed the experts’ assignments in achieving the objectives and in execution time. Experts agree that the generated schedules can give good management suggestions and work as an auxiliary schedule for managers. Since our model considers more factors such as learning, communication overhead, skill fitness, managers can also take the generated results to inspect their management options.

## Empirical Study and Discussion

To evaluate the effectiveness and performance of our approach, several problems with different sizes and constraints were designed and conducted. Analysis and discussion of the simulation results are reported.

### Effectiveness of Our Approach

To confirm the effectiveness of our approach, smaller experiments were designed. It is a project with 4 tasks with task properties and employee properties in Tables 8 and 9.

**Table 8:** Task Properties of a Project with 4 Tasks and 3 Employees.

Task No.	Estimated Effort	Deadline	Penalty Per Day	Precedence Tasks	Required Skills
Design	0.1	Jan-15	20000		Analysis C++ Word
Programming1	0.1	Jan-15	10000	1	Analysis C++
Programming2	0.2	Jan-15	10000	1	Analysis C++
Documentation	0.2	Jan-15	10000	2,3	Word

**Table 9:** Employee Properties of a Project with 4 Tasks and 3 Employees.

EmpID	Hourly Salary	Max Workload	Learning	Analysis	C++	Word
1	15	100%	1.25	5	3	2
2	15	100%	1.15	4	4	4
3	18	100%	1.45	2	5	2

We also get good results in this simple example. The result from the GA-HC is shown in Table 10. After the soft deadline is changed to Jan. 10, the result in Table 11 shows that employees are assigned to the work more to get things done more quickly. By comparing these two results, the difference is that Employee 1 is also assigned to Task 3 and the execution time for Task 3 decreases from 6 days to 3 days. It can decrease the penalty cost by finishing tasks earlier. Since even Employee 1 is added to the team for Task 1, the time to finish tasks can only be decreased a little bit but not enough to decrease it from 3 days to 2 days. Therefore, Employee 2 and Employee 3 are considered as the best solution for Task 1 in this situation. Choosing Employee 3 for Task 1 and Task 4 instead of Employee 1 because Employee 3 has higher learning factor than Employee 1. From the above analysis, we can see the correctness of our model in some aspects.

**Table 10:** Generated Schedule for a Simple Project of 4 Tasks.

Task ID	Days	Start Date	End Date	*Resource*
Design	3	Jan 1	Jan 3	Employee2, Employee3
Programming 1	5	Jan 4	Jan 8	Employee2
Programming 2	5	Jan 4	Jan 8	Employee1, Employee3
Documentation	6	Jan 9	Jan 14	Employee2, Employee3

**Table 11:** Generated Schedule for a Simple Project of 4 Tasks with More Tight Deadline.

Task ID	Days	Start Date	End Date	*Resource*
Design	3	Jan 1	Jan 3	Employee2, Employee3
Programming 1	5	Jan 4	Jan 8	Employee2
Programming 2	5	Jan 4	Jan 8	Employee1, Employee3
Documentation	3	Jan 9	Jan 12	Employee1, Employee2, Employee3

### Performance of Optimization Approach

To validate our approach, 10 simplified project management problems are designed with the objective to “Find a valid schedule that has lowest money cost, irrespective of other factors, such as learning, overwork”. In such situations, any optimum solution should follow the rules that any available employees with lowest salary rate would be firstly assigned to a task. The GA parameter is the same as the previous section. All the results from the experiments obey our previous projections. We also design some moderately constrained problems, highly constrained problems, very small problems and large problems generated by simulation data. To find the GA-HC performance in those different examples, comparison experiments are conducted. The mean and standard deviation (*σ*) of the result data are reported in Table 12 which shows that the GA-HC performance in the relative big problem (21 tasks, 10 employees) is good since all the results are close, while results are in a wider range in relatively simple problems.

**Table 12:** GA-HC Performance in a Relative Large Problem (21 Tasks, 10 Employees) and Small Problem (4 Tasks, 3 Employees).

Cost (RMB)	Mean	*σ*
Project A (21 tasks, 10 employees)	130311	4200
Project B (4 tasks, 3 employees)	5218	690
Project C (4 tasks, 3 employees with more tight constraints)	39085	1235

A number of researches have defined certain kinds of problems that GAs work better than other heuristic methods and the criteria to compare different problems and algorithms. Mostly the comparison focuses on GAs and HC algorithms [41]. To compare the performance between different heuristic methods, we chose Steady GA, Hill Climbing and Steady GA with HC on our model based on the criteria of the quality of the best, mean, worst solution from different algorithm. From the results shown in Tables 13–15 in large, medium and small cases, the steady GA and the GA-HC outperform the HC in most cases. Usually a HC algorithm is much faster than a GA. However, the landscape in our problem has many local optima which makes a HC algorithm difficult to achieve the global optimum. So in big cases, GAs outperforms HC algorithms on finding good quality solutions when the problems are becoming complicated, as is the case for many software projects. The experiment case consists of 30 tasks for which 20 employees were available. The employees, in turn, each possessed 5 skills to a greater or lesser extent. Each of the 5 skills was needed by at least one task and many tasks required multiple skills. The best cost computed by HC is 136760 with 1000 initial individuals in the population while the cost by the steady GA is 127448 which outperformed the best fitness achieved by HC, where lower number means lower cost and is better. In a smaller case of 15 tasks, 10 employees in which the GA outperforms the HC dramatically, and in a case of 8 tasks, 5 employees which do not show much difference in the two methods but the distribution of solutions from the HC is bigger than the GA in our experiment. Combining the GA’s ability on global optimization and the HC’s ability on local optimization, the performance of the GA and the GA-HC in a relative larger project scheduling problem shows similar result while GA converges very slowly in later stage and the running time of the GA-HC outperforms the GA apparently. When the project size increases, the calculation time of the GA could take several hours which becomes a burden to managers. Overall, the GA-HC is generally a good choice in the software project scheduling circumstance.

**Table 13:** Comparison between GA, HC, GA-HC in a Project of 30 Tasks and 20 Employees.

Cost(RMB)	Best	Mean	Worst	Average Time
Steady GA (1000 generations)	127448	133019	142670	35m
Hill Climbing	136760	174203	256745	10m
Steady GA (500 generations) with HC	127448	133346	137023	19m

**Table 14:** Comparison between GA, HC, GA-HC in a Project of 15 Tasks and 10 Employees.

Cost(RMB)	Best	Mean	Worst	Average Time
Steady GA (1000 generations)	38956	39822	43112	20m
Hill Climbing	44200	46297	54002	1m
Steady GA (500 generations) with HC	34248	39889	42344	12m

**Table 15:** Comparison between GA, HC, GA-HC in a Project of 8 Tasks and 5 Employees.

Cost(RMB)	Best	Mean	Worst	Average Time
Steady GA (600 generations)	5460	5580	5736	60s
Hill Climbing	5504	6299	8686	3s
Steady GA (300 generations) with HC	5480	5590	5700	28s

### Study on Dynamic Factors

Table 16 shows the calculated costs with certain factors excluded. Without the learning factors or communication overhead, the overall cost increases as expected. Without schedule pressure, no solution is found in this highly constrained case. Although these results are intuitive in this simple scenario, in a more complicated situation data can help to analyze the influence of certain factors and to assist managers to make more sensible decisions.

**Table 16:** Comparison of Schedule Costs with/without Factors.

Cost(RMB)	Small Case	Medium Case	Large Case
With all the factors	4690	13110	234335
With only learning factor	6003	15117	285356
With only communication overhead factor	4200	12601	201314
With only schedule pressure factor	5230	13567	244500
Without all the factors	n/a	18670	342034

## Conclusions and Future Work

The main purpose of the model developed in this work is to assist managers in determining the best resource allocation. The unpredictable factors influencing a software development project are too many and complicate planning problems. This paper proposes a team productivity-based model to support generating project schedule. Our model is especially for modeling dynamic factors related to staff. A new genome of the GA is designed for the proposed model. It overcomes the complexities by generating only valid solutions in search space and decreases the computation burden. Additionally, HC is designed to make further efforts to alleviate the computation burden of the GA but achieve same quality of results. We also propose a software project rescheduling approach considering efficiency and stability. This approach is based on formulating software project scheduling and rescheduling situation as an optimization problem via a genetic algorithm. The proposed method will help a manager to do scheduling with the option he made to put the project back on track. Experiments and simulation results demonstrate that it has the ability to produce valid schedule and reschedule alternatives. Case studies with comparison to schedules generated by project management experts prove that it could provide reasonable decision-making support for managers.

Still, there are areas that can be improved. When formulating software project scheduling and rescheduling situation as an optimization problem via a genetic algorithm, studies should be directed to balance the parameters of the objective function in different situations. Evaluations of possible impact of all the available control options such as adding more people or leaving a project lag behind could be integrated. In rescheduling, sensitivity study of the parameters of stability and efficiency should be directed to balance the effect of stability and efficiency in different situations. In addition, evaluations of possible impact of all the available control options should be integrated in the model to support control decision making in software process. Currently the simulation is not taking into account the interactions between parallel tasks. For example, if an employee is assigned to two tasks at the same time, the factors relating to both tasks (such as *f*_*learning*_) are calculated separately in each task. The issue is our future work and needs to be further studied. For more accurate estimation in project planning, tuning our simulation models is extremely important. The process of judging the validity of a model should be conducted, such as extreme condition test to test whether the model behaves reasonably under extreme conditions or extreme policies. Sensitive analysis could also be applicable for parameter tuning. Finally, case studies are necessary and essential to evaluate the performance of our work. After the models have been completely established, case studies will help customize these models to a specific organization when needed. Integration with current commercial software tools can help transfer current advanced techniques such as what our research group developed into industrial use.

## Supporting Information

S1 Input Input XML File for Boyuan Software Company Case. This XML file is the input for the software *Sched-SPM*. The project is from Boyuan Software Company and consists of 19 tasks and 9 employees.
(XML)

S2 Input Input XML File for Web Site Case. This XML file is the input for the software *Sched-SPM*. The project is from a web site development case and consists of 28 tasks and 10 employees.
(XML)

S3 Input Input XML File for Project A. This XML file is the input for the software *Sched-SPM*. Project A consists of 21 tasks and 10 employees.
(XML)

S4 Input Input XML File for Project B. This XML file is the input for the software *Sched-SPM*. Project B consists of 4 tasks and 3 employees.
(XML)

S5 Input Input XML File for Project C. This XML file is the input for the software *Sched-SPM*. Project C consists of 4 tasks and 3 employees with more tight constraints.
(XML)

S6 Input Input XML File for Project of 8 Tasks and 5 Employees. This XML file is the input for the software *Sched-SPM* for a project of 8 tasks and 5 employees.
(XML)

S7 Input Input XML File for Project of 15 Tasks and 10 Employees. This XML file is the input for the software *Sched-SPM* for a project of 15 tasks and 10 employees.
(XML)

S8 Input Input XML File for Project of 30 Tasks and 20 Employees. This XML file is the input for the software *Sched-SPM* for a project of 30 tasks and 20 employees.
(XML)

S1 Output Output XML File for Boyuan Software Company Case. This XML file is the best result generated by the software *Sched-SPM* for the Boyuan Software Company case.
(XML)

S2 Output Output XML Files for Web Site Case. These XML files are generated by the software *Sched-SPM* for the web site case.
(RAR)

S3 Output Output XML Files for Project A. These XML files are generated by the software *Sched-SPM* for Project A.
(RAR)

S4 Output Output XML Files for Project B. These XML files are generated by the software *Sched-SPM* for Project B.
(RAR)

S5 Output Output XML Files for Project C. These XML files are generated by the software *Sched-SPM* for Project C.
(RAR)

S6 Output Output XML Files by GA for Project of 8 Tasks and 5 Employees. These XML files are generated by GA for a project of 8 tasks and 5 employees.
(RAR)

S7 Output Output XML Files by GA-HC for Project of 8 Tasks and 5 Employees. These XML files are generated by GA-HC for a project of 8 tasks and 5 employees.
(RAR)

S8 Output Output XML Files by HC for Project of 8 Tasks and 5 Employees. These XML files are generated by HC for a project of 8 tasks and 5 employees.
(RAR)

S9 Output Output XML Files by GA for Project of 15 Tasks and 10 Employees. These XML files are generated by GA for a Project of 15 Tasks and 10 Employees.
(RAR)

S10 Output Output XML Files by GA-HC for Project of 15 Tasks and 10 Employees. These XML files are generated by GA-HC for a project of 15 tasks and 10 employees.
(RAR)

S11 Output Output XML Files by HC for Project of 15 Tasks and 10 Employees. These XML files are generated by HC for a project of 15 tasks and 10 employees.
(RAR)

S12 Output Output XML Files by GA for Project of 30 Tasks and 20 Employees. These XML files are generated by GA for a project of 30 tasks and 20 employees.
(RAR)

S13 Output Output XML Files by GA-HC for Project of 30 Tasks and 20 Employees. These XML files are generated by GA-HC for a project of 30 tasks and 20 employees.
(RAR)

S14 Output Output XML Files by HC for Project of 30 Tasks and 20 Employees. These XML files are generated by HC for a project of 30 tasks and 20 employees.
(RAR)

S1 MS Project File Result by Expert 1 for Boyuan Software Company Case—Scheduling. This MS project file is the scheduling result from Expert 1 for the Boyuan Software Company case.
(MPP)

S2 MS Project File Result by Expert 1 for Boyuan Software Company Case—Rescheduling. This MS project file is the rescheduling result from Expert 1 for the Boyuan Software Company case.
(MPP)

S3 MS Project File Result by Expert 2 for Boyuan Software Company Case—Scheduling. This MS project file is the scheduling result from Expert 2 for the Boyuan Software Company case.
(MPP)

S4 MS Project File Result by Expert 2 for Boyuan Software Company Case—Rescheduling. This MS project file is the rescheduling result from Expert 2 for the Boyuan Software Company case.
(MPP)

S5 MS Project File Result by Expert 1 for Web Site Case. This MS project file is the scheduling result from Expert 1 for the web site development case.
(MPP)

S6 MS Project File Result by Expert 2 for Web Site Case. This MS project file is the scheduling result from Expert 2 for the web site development case.
(MPP)

## References

[1] Chang CK, Christensen MJ, Zhang T. Genetic Algorithms for Project Management. Annal of Software Engeering. 2001;11(1):107–139. 10.1023/A:1012543203763

[2] Chao C. Software Project Management Net: a New Methodology on Software Management. PhD dissertation, University of Illinois at Chicago, Department of Electrical Engineering and Computer Science, 1994.

[3] Chang CK, Jiang Hy, Di Y, Zhu D, Ge Y. Time-line Based Model for Software Project Scheduling with Genetic Algorithms. Information and Software Technology. 2008;50(11):1142–1154. 10.1016/j.infsof.2008.03.002

[4] Alba E, Francisco Chicano J. Software Project Management with GAs. Information Sciences. 2007;177(11):2380–2401. 10.1016/j.ins.2006.12.020

[5] Gueorguiev S, Harman M, Antoniol G. Software Project Planning for Robustness and Completion Time in the Presence of Uncertainty Using Multi Objective Search Based Software Engineering. In: Proceedings of the 11th Annual Conference on Genetic and Evolutionary Computation. GECCO’09. New York, NY, USA: ACM; 2009. p. 1673–1680.

[6] Plekhanova V. On Project Management Scheduling Where Human Resource is a Critical Variable. In: Proceedings of the 6th European Workshop on Software Process Technology. EWSPT’98. London, UK: Springer-Verlag; 1998. p. 116–121.

[7] Lederer AL, Prasad J. Causes of Inaccurate Software Development Cost Estimates. Journal of Systems & Software. 1995;31(2):125–134. 10.1016/0164-1212(94)00092-2

[8] Hartmann S, Kolisch R. Experimental evaluation of state-of-the-art heuristics for the resource-constrained project scheduling problem. European Journal of Operational Research. 2000;127(2):394–407. 10.1016/S0377-2217(99)00485-3

[9] Holland JH. Adaptation in Natural and Artificial Systems. Ann Arbor, MI, USA: University of Michigan Press; 1975 Available from: http://books.google.com/books?id=YE5RAAAAMAAJ.

[10] Contreras-Bolton C, Parada V. Automatic Combination of Operators in a Genetic Algorithm to Solve the Traveling Salesman Problem. PLoS ONE. 2015;10(9). 10.1371/journal.pone.0137724 PMC4569577 26367182

[11] Harman M, Mansouri SA, Zhang Y. Search-based Software Engineering: Trends, Techniques and Applications. ACM Comput Survey. 2012;45(1):11:1–11:61. 10.1145/2379776.2379787

[12] Ferrucci F, Harman M, Ren J, Sarro F. Not Going to Take This Anymore: Multi-objective Overtime Planning for Software Engineering Projects. In: Proceedings of the 2013 International Conference on Software Engineering. ICSE’13. Piscataway, NJ, USA: IEEE Press; 2013. p. 462–471.

[13] Ren J, Harman M, Penta MD. Cooperative Co-evolutionary Optimization of Software Project Staff Assignments and Job Scheduling. In: Proceedings of the Third International Conference on Search Based Software Engineering. SSBSE’11. Berlin, Heidelberg: Springer-Verlag; 2011. p. 127–141.

[14] Pfeiffer A, Kadar B, L M. Stability-oriented Evaluation of Hybrid Rescheduling Methods in a Job-shop With Machine Breakdowns. In: Proceedings of the 39th CIRP ISMS; 2006. p. 173–178.

[15] Cowling P, Johansson M. Using real time information for effective dynamic scheduling. European Journal of Operational Research. 2002;139(2):230–244. 10.1016/S0377-2217(01)00355-1

[16] Katragjini Prifti K, Vallada Regalado E, Ruiz García R. Stability-oriented Evaluation of Rescheduling Strategies, by Using Simulation. International Journal of Production Research. 2013;51(3):780–797.

[17] Sukhodolsky J. Optimizing Software Process Control. SIGSOFT Softw Eng Notes. 2001;26(2):59–63. 10.1145/505776.505791

[18] Boehm BW. Software Engineering Economics. 1st ed. Upper Saddle River, NJ, USA: Prentice Hall PTR; 1981.

[19] Boehm BW, Abts C, Brown AW, Chulani S, Clark BK, Horowitz E, et al Software Cost Estimation with COCOMO II. 1st ed. Upper Saddle River, NJ, USA: Prentice Hall PTR; 2000.

[20] Azzeh M, Neagu D, Cowling PI. Analogy-based Software Effort Estimation Using Fuzzy Numbers. Journal of Systems and Software. 2011;84(2):270–284. 10.1016/j.jss.2010.09.028

[21] Chiu NH, Huang SJ. The Adjusted Analogy-based Software Effort Estimation Based on Similarity Distances. Journal of Systems and Software. 2007;80(4):628–640. 10.1016/j.jss.2006.06.006

[22] Pendharkar PC, Subramanian GH, Rodger JA. A Probabilistic Model for Predicting Software Development Effort. IEEE Transaction of Software Engineering. 2005;31(7):615–624. 10.1109/TSE.2005.75

[23] Chavoya A, Lopez-Martin C, Andalon-Garcia I, Meda-Campaña M. Genetic Programming as Alternative for Predicting Development Effort of Individual Software Projects. PLoS ONE. 2012;7(11). 10.1371/journal.pone.0050531 PMC3511534 23226305

[24] Abdel-Hamid T, Madnick SE. Software Project Dynamics: An Integrated Approach. Upper Saddle River, NJ, USA: Prentice-Hall, Inc; 1991.

[25] Barros M, Werner C, Travassos G. System Dynamics Extension Modules for Software Process Modeling. In: Proceedings of 2003 Software Process Simulation and Modeling Workshop. ProSim’03; 2003.

[26] Pfahl D, Lebsanft K. Integration of System Dynamics Modeling with Descriptive Process Modeling and Goal-oriented Measurement. Journal of Systems and Software. 1999;46(2/3):135–150. 10.1016/S0164-1212(99)00007-2

[27] Hansen GA. Simulating Software Development Processes. Computer. 1996;29(1):73–77. 10.1109/2.481468

[28] Kouskouras KG, Georgiou AC. A Discrete Event Simulation Model in the Case of Managing a Software Project. European Journal of Operational Research. 2007;181(1):374–389. 10.1016/j.ejor.2006.05.031

[29] Lakey PB. A Hybrid Software Process Simulation Model for Project Management. In: Proc. 2003 Software Process Simulation and Modeling Workshop. ProSim’03; 2003.

[30] Martin RH, Raffo D. A model of the software development process using both continuous and discrete models. Software Process: Improvement and Practice. 2000;5(2–3):147–157. 10.1002/1099-1670(200006/09)5:2/3<147::AID-SPIP122>3.0.CO;2-T

[31] Donzelli P. A Decision Support System for Software Project Management. IEEE Software. 2006;23(4):67–75. 10.1109/MS.2006.90

[32] Penta Md, Harman M, Antoniol G, Qureshi F. The Effect of Communication Overhead on Software Maintenance Project Staffing: a Search-Based Approach. In: 2007 IEEE International Conference on Software Maintenance. IEEE Press; 2007. p. 315–324.

[33] Shoham I, McIvor A. OpenSched; 2001-2006. http://opensched.sourceforge.net/.

[34] Stylianou C, Gerasimou S, Andreou AS. A Novel Prototype Tool for Intelligent Software Project Scheduling and Staffing Enhanced with Personality Factors. In: 2012 IEEE 24th International Conference on Tools with Artificial Intelligence. vol. 1; 2012. p. 277–284.

[35] Ventana Systems Inc. Vensim Software Tool; 2015. Available: http://vensim.com/.

[36] Herroelen W, Leus R. Project scheduling under uncertainty: Survey and research potentials. European Journal of Operational Research. 2005;165(2):289–306. 10.1016/j.ejor.2004.04.002

[37] Ge Y. Software Project Rescheduling with Genetic Algorithms. In: Artificial Intelligence and Computational Intelligence, 2009. AICI’09. International Conference on. vol. 1; 2009. p. 439–443.

[38] Gen M, Cheng R. Genetic Algorithms and Engineering Optimization. New York, NY, USA: John Wiley & Sons; 2000.

[39] Wall M. GALib; 1996-2007. Available: http://lancet.mit.edu/ga/.

[40] Di Y. Timeline Based Model For Job Scheduling with Genetic Algorithms. Master’s thesis, University of Illinois at Chicago, Department of Electrical Engineering and Computer Science, 2001.

[41] Mitchell M, Holland JH, Forrest S. When will a Genetic Algorithm Outperform Hill Climbing. In: Cowan JD, Tesauro G, Alspector J, editors. Advances in Neural Information Processing Systems 6. San Francisco, CA: Morgan-Kaufmann; 1994. p. 51–58.

